# Ocular Ultrasonography in Healthy Calves with Different Transducers

**DOI:** 10.3390/ani13040742

**Published:** 2023-02-19

**Authors:** Giuliano Borriello, Flaminia Valentini, Mauro Rampinelli, Sara Ferrini, Giulia Cagnotti, Antonio D’angelo, Claudio Bellino

**Affiliations:** Department of Veterinary Science, University of Turin, 10095 Grugliasco, Italy

**Keywords:** calves, welfare, prevention, ultrasonography

## Abstract

**Simple Summary:**

Ocular ultrasonography for eye examination is seldom performed in cattle. Despite this, some ocular pathologies have detrimental effects on animal welfare. Thus, the knowledge of normal ultrasonographic eye patterns and biometry is helpful for correct diagnosis. In this study, we compared the measurements made with three ultrasound transducers (linear, convex, transrectal gynecological) in field conditions on 20 healthy calves (10 Holstein breed, 10 Piedmontese breed). While both linear and transrectal gynecological transducers can be used for eye examination in the field, widespread application of the transrectal gynecological probe make it the most suitable choice for ocular ultrasonography in field conditions.

**Abstract:**

Ocular ultrasonography is seldom performed in cattle. Here, we compared three ultrasound probes (linear, convex, transrectal gynecological) for the measurement of eight eye biometry parameters on vertical and horizontal scans. The sample population was 20 healthy calves (n = 10 Holstein, n = 10 Piedmontese breed). Intragroup (same probe for vertical vs. horizontal scanning) and intergroup (different probes measuring the same biometric parameter) comparisons were performed using Student’s *t*-test and the Mann–Whitney *U* test. Statistical significance was set at *p* ≤ 0.05. Intragroup comparison revealed few significant differences. Ultrasound examination with the convex transducer detected more differences than either of the two other probes on vertical (linear six out of eight; transrectal gynecological four out of eight) and horizontal (linear six out of eight; transrectal gynecological six out of eight) scans. Similar results were obtained for both breeds. More non-valuable parameters on the horizontal (77 out of 320, 24%) and the vertical (85 out of 320, 26%) (*p* ≤ 0.001) scans were obtained with the convex transducer. Both linear transducers were found comparable for ocular ultrasonography in field conditions. However, given its widespread application in the field, the transrectal gynecological transducer may offer veterinarians the added advantage of familiarity and ease-of-use without any additional costs.

## 1. Introduction

Ophthalmic ultrasonography is widely used in human and veterinary medicine for studying ocular biometry, eye exams and emergency medicine [1,2]. Cattle, as other animals, are affected by various eye diseases, both primary (i.e., infectious bovine keratoconjunctivitis) and secondary (i.e., uveitis), capable to impair cattle’s welfare and production at both the individual and herd level [3]. Calves, in particular, are susceptible to various eye lesions, including uveitis, endophthalmitis, and hypopyon, which can alter the normal structure of the eye [4]. Although these lesions could be clinically obvious, in their early phases, they may not be easily distinguishable from congenital diseases, such as congenital cataracts, and the impairment of ocular structures could be non-reversible. Thus, eye ultrasonography may provide a diagnostic tool for correct diagnosis and the right, early, therapeutic approach [4]. Knowledge of normal ultrasonographic eye patterns and biometry in healthy animals is essential for correct diagnosis [3]. However, few studies have been performed in cattle [5,6]. Thus, considering the importance of the calf for the productivity of the farm and the widespread use of ultrasound equipment in the field practice, the aim of this study was (i) to provide a description of the non-pathological ultrasonographic features of the calf eye using different ultrasonographic transducers in Holstein and Piedmontese calves, and (ii) to asses the most suitable ultrasound transducer for on-farm visits.

## 2. Materials and Methods

The study was conducted between April 2021 and January 2022 on six farms in northern Italy (Piedmont). Inclusion criteria were:

- Age < 30 days
- Clinically healthy
- No detectable ocular lesions.

Clinical and eye examinations were performed by an experienced operator. The eye exam entailed an evaluation of eyesight, menace reaction, pupillary response, eyelid reflex, nystagmus, strabismus, proptosis, enophthalmos, and tearing [7]. The data were recorded for further analysis. Animal owners were informed about the procedures and the potential risks before obtaining permission for examination. Ultrasound examination was carried out on the farm, with the animals in standing position, not sedated, and handled by a single operator. Ultrasound with three different transducers was performed by an expert operator using an Esaote MyLab©One Vet linear (LT) (4–13 MHz), convex (CxT) (1–8 MHz), and transrectal gynecological (LgT) (5–10 MHz) transducer. The eye region was not shaved. Transpalpebral examination was performed after application of the ultrasound gel. For each eye, horizontal and vertical imaging planes were obtained with each type of transducer and at least one image of each scan was recorded. At the end of each exam, the effect of the technique on animal welfare was assessed for local side effects and interaction between the calf and the environment and the operators.

Ocular biometry was evaluated on horizontal and vertical scans and measured for the following parameters [3]:

- Axial length of the globe (AL): distance from the mid-cornea to the posterior pole of the globe including the scleroretinal rim;
- Depth of the anterior chamber (DAC): distance from the mid-cornea to the anterior lens capsule;
- Vitreous depth (VD): distance from the lens to the scleroretinal rim;
- Corneal thickness (CT): distance from the anterior to the posterior corneal epithelium;
- Scleroretinal rim thickness (SRT): distance from the internal epithelium to the external scleroretinal rim;
- Lens length (LL): distance from the anterior to the posterior chamber of the lens;
- Lens thickness (LTK): distance between the equatorial poles of the lens;
- Optic nerve diameter (OD).

Statistical analysis was performed on the whole sample and separately on the two breeds. Differences in biometric measurement between the horizontal and the vertical scan taken with the same probe were compared. Differences in eye biometry were similarly compared. Data analysis was performed using R version 4.1.3 (R Project for Statistical Computing, R Core Team (2022). R Foundation for Statistical Computing, Vienna, Austria. https://www.R-project.org/). Normal distribution was evaluated using the Shapiro–Wilk test. Data are expressed as mean ± standard deviation (±SD) or median, minimum, and maximum. Ocular parameters were compared between breeds and for the whole sample using Student’s *t*-test or the Mann–Whitney *U* test. Statistical significance was set at *p* ≤ 0.05.

## 3. Results

The study sample was 20 female clinically healthy calves of Holstein (n = 10) or Piedmontese (n = 10) breed. The median age of the entire sample was 8 days (range, 2–30), 5 days (range, 2–18) for Holstein and 12 days (range, 6–30) for Piedmontese breed. Handling was well tolerated by the animals and the procedure took 1:25–4:00 min to complete. Ultrasound using the CxT took more time than with the other two probes (LT: 02:14 ± 0:28 min; LgT: 02:04 ± 0:26 min) (*p* < 0.01) in the whole sample (03:10 ± 0:33 min). Similarly, with the convex transducer, more time was also spent in the Holstein (CxT: 03:16 ± 0:33 min; LT: 02:16 ± 00:25 min; LgT: 02:08 ± 00:26 min; *p* ≤ 0.01) and in the Piedmontese calves than with other two probes (CxT: 03:34 ± 0:34 min; LT: 02:12 ± 0:31; LgT: 01:54 ± 00:26; *p* ≤ 0.01). All animals resumed normal activity within a few minutes after examination. No signs of ocular or systemic discomfort were noted.

### 3.1. Entire Sample

**Figure 1:**
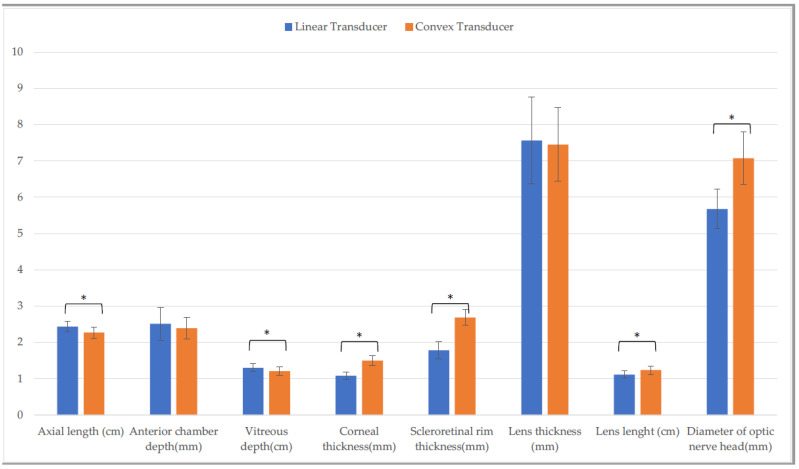
Comparison of biometric parameters measured with the linear and the convex transducer in the entire sample. Horizontal scan. (*: *p* ≤ 0.001).

Data for the entire sample are reported in the Supplementary File (Table S1). No significant differences were found between the horizontal and the vertical scans obtained with each probe. A comparison of biometrics showed differences between the CxT and the LT probe on the horizontal scans (Figure 1) for axial length, vitreous depth, corneal thickness, scleroretinal rim thickness, lens length, and diameter of optic nerve head (*p* ≤ 0.001).

**Figure 2:**
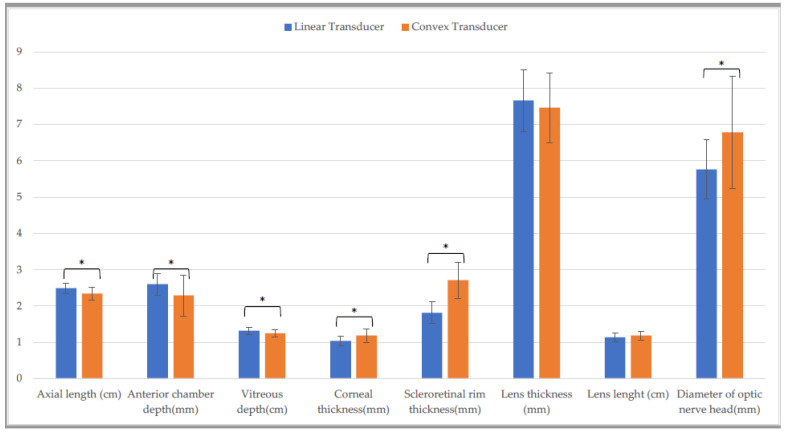
Comparison of biometric parameters measured with the linear and the convex transducer in the entire sample. Vertical scan (*: *p* ≤ 0.001).

In addition, CxT differed from LT for six structures (Figure 2) in vertical scan: axial length, anterior chaber depth, vitreous depth, corneal thickness, scleroretinal rim thickness, diameter of optic nerve head (*p* ≤ 0.001).

**Figure 3:**
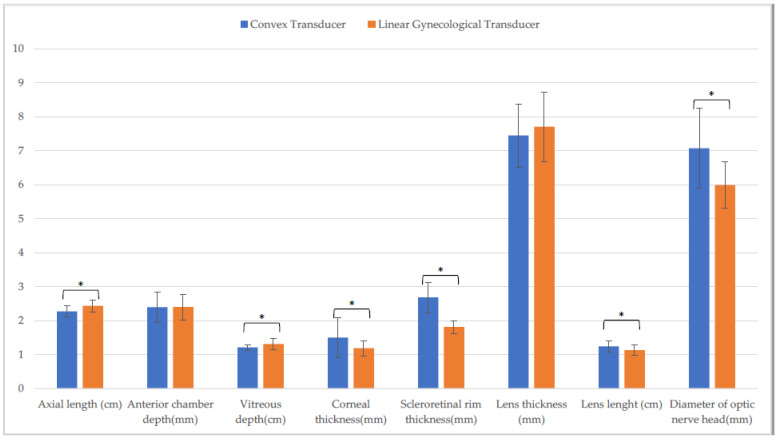
Comparison of biometric parameters measured with the convex and the linear gynecological transducer in the entire sample. Horizontal scan. (*: *p* ≤ 0.001).

**Figure 4:**
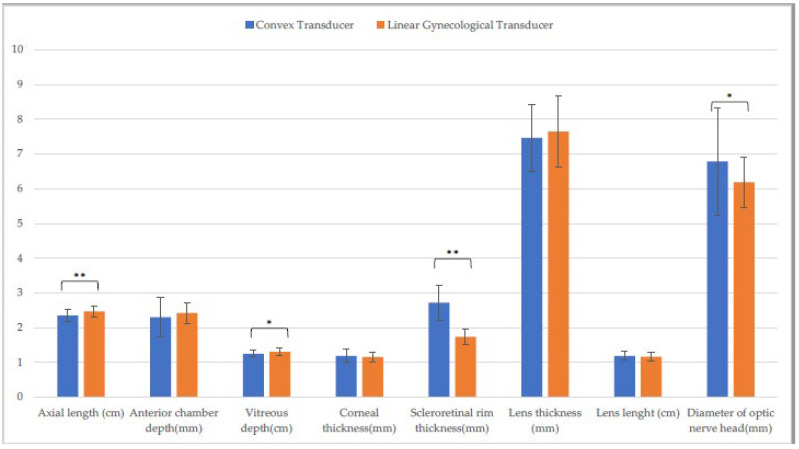
Comparison of biometric parameters measured with the convex and the linear gynecological transducer in the entire sample. Vertical scan. (*: *p* ≤ 0.05; **: *p* ≤ 0.01).

Differences in CxT and LgT measurements on the horizontal (Figure 3) and the vertical scan (Figure 4) were found. On the horizontal scan, there were differences between CxT and LgT measurement of axial length, anterior chamber depth, vitreous depth, corneal thickness, scleroretinal rim thickness, and optic nerve diameter (*p* ≤ 0.001). There were also differences between CxT and LgT measurement of anterior chamber depth, scleroretinal rim thickness (*p* ≤ 0.001), vitreous depth, and diameter of optic nerve head (*p* ≤ 0.05).

Comparing LT and LgT in horizontal scan, statistical differences were recorded for two structures: corneal thickness (*p* ≤ 0.001), optic nerve diameter (*p* ≤ 0.05). Instead, in vertical scan, differences between LT and LgT were observed for three structures: anterior chamber depth (*p* ≤ 0.001), corneal thickness (*p* ≤ 0.001), optic nerve diameter (*p* ≤ 0.05). No differences between horizontal and vertical scans were found for LgT and LT. No other differences between LgT and LT measurement on the horizontal and the vertical scan were found.

### 3.2. Holstein Calves

Table 1 presents the biometric parameters measured in the Holstein calves. There were statistically significant differences in lens length (*p* ≤ 0.001) and corneal thickness (*p* ≤ 0.05) between the horizontal and the vertical scan made with the CxT.

**Figure 5:**
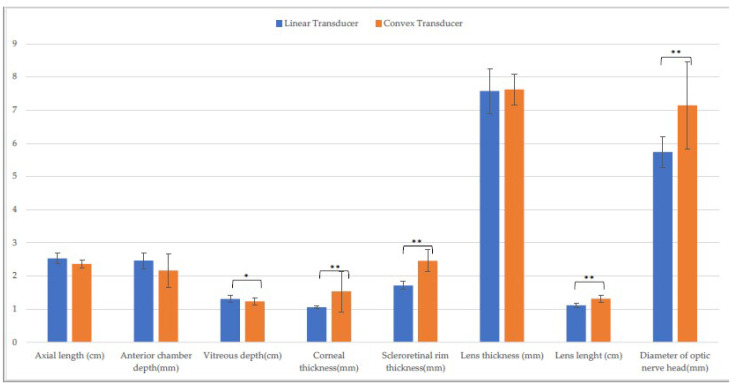
Comparison between linear and convex transducers in Holstein calves. Horizontal scan. (*: *p* ≤ 0.01; **: *p* ≤ 0.001).

**Figure 6:**
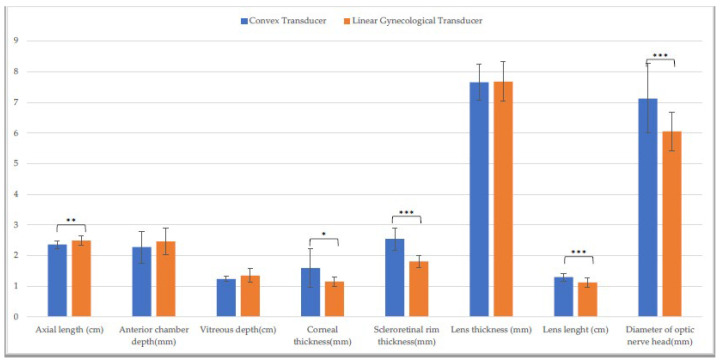
Comparison between convex and linear gynecological transducers in Holstein calves. Horizontal scan. (*: *p* ≤ 0.05; **: *p* ≤ 0.01; ***: *p* ≤ 0.001).

Measurement of vitreous depth, corneal thickness, scleroretinal rim thickness, lens length, and optic nerve diameter differed between the CxT and the LT on the horizontal scan (*p* ≤ 0.001), whereas on the vertical scan measurement they differed for axial length, scleroretinal rim thickness (*p* ≤ 0.001), and corneal thickness (*p* ≤ 0.05). There were differences in axial length, scleroretinal rim thickness, lens length, optic nerve diameter (*p* ≤ 0.001), and corneal thickness (*p* ≤ 0.05) between the CxT and the LgT on the horizontal scans (Figure 5), whereas on the vertical scans there was a difference in axial length (*p* ≤ 0.05) and scleroretinal rim thickness (*p* ≤ 0.001) between the CxT and the LgT (Figure 6).

Comparing LT and LgT in horizontal scan, statistical differences were recorded only for two structures: corneal thickness and optic nerve diameter (*p* ≤ 0.001). Moreover, in vertical scan, differences were observed for two structures: anterior chamber depth (*p* ≤ 0.05), corneal thickness (*p* ≤ 0.001). No differences between horizontal and vertical scans were found for LgT and LT.

### 3.3. Piedmontese Calves

Table 2 presents the biometric parameters measured in the Piedmontese calves. There were differences in axial length measured with the CxT on the horizontal and the vertical scans (*p* ≤ 0.001). There were no differences in LgT measurement between the horizontal and the vertical scans, whereas there was a difference in scleroretinal rim thickness measured with the LT between the horizontal and the vertical scans (*p* ≤ 0.05).

**Figure 7:**
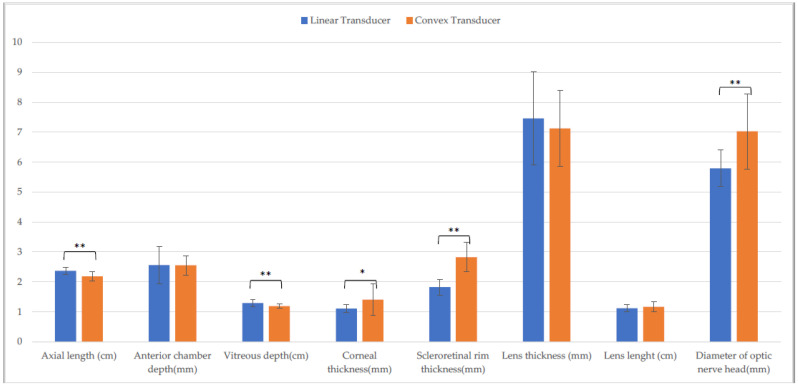
Comparison of ocular biometry with the linear and the convex transducer in Piedmontese calves. Horizontal scan. (*: *p* ≤ 0.05; **: *p* ≤ 0.01).

**Figure 8:**
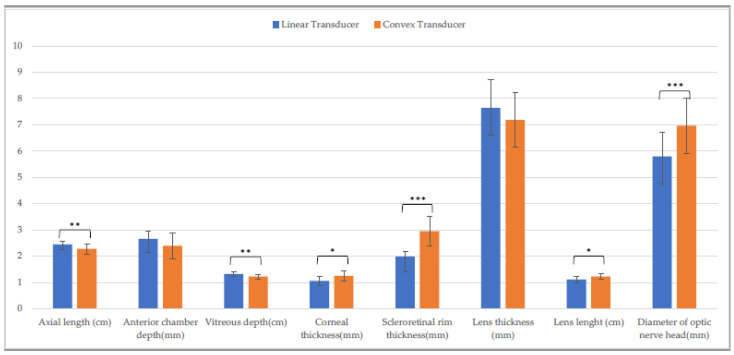
Comparison of ocular biometry with the linear and the convex transducer in Piedmontese calves. Vertical scan. (*: *p* ≤ 0.05; **: *p* ≤ 0.01; ***: *p* ≤ 0.001).

There was a difference in axial length, vitreous depth, scleroretinal rim thickness, optic nerve diameter (*p* ≤ 0.001), and corneal thickness (*p* ≤ 0.05) between the CxT and the LT on the horizontal scan (Figure 7), whereas the measurement of axial length, vitreous depth, scleroretinal rim thickness, optic nerve diameter (*p* ≤ 0.001), corneal thickness, and lens length differed between the CxT and the LT on the vertical scan (*p* ≤ 0.05) (Figure 8).

**Figure 9:**
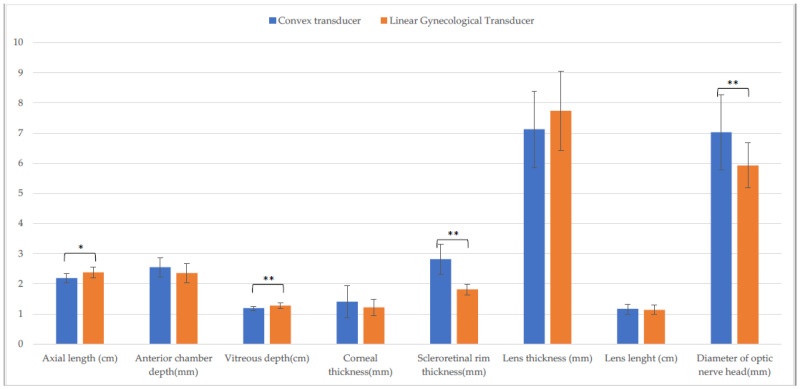
Comparison of ocular biometry with the linear and the convex transducer in Piedmontese calves. Horizontal scan. (*: *p* ≤ 0.05; **: *p* ≤ 0.01).

Similarly, comparing CxT and LgT in horizontal scanning, four structures were statistically different (Figure 9): axial length (*p* ≤ 0.01), vitreous depth, scleroretinal rim thickness, optic nerve diameter (*p* ≤ 0.001). Moreover, in vertical scanning, CxT differed from LgT for four structures: axial length, vitreous depth (*p* ≤ 0.05), scleroretinal rim thickness, and optic nerve diameter (*p* ≤ 0.001).

Instead, comparing LT and LgT in vertical scan, statistical differences were recorded for two structures: corneal thickness and scleroretinal rim thickness (*p* ≤ 0.001). Meanwhile, in horizontal scan, differences were observed just for corneal thickness (*p* ≤ 0.001)

### 3.4. Comparison between Breeds

Measurement of axial length with the LT was greater in the Holstein calves on the horizontal and vertical scans (*p* ≤ 0.001), whereas scleroretinal rim thickness was greater in the Piedmontese calves on the vertical scans. Measurement of axial length with the CxT was significantly greater in the Holstein calves on the horizontal and the vertical scans (*p* ≤ 0.001), whereas scleroretinal rim thickness was greater in the Piedmontese calves (*p* ≤ 0.05). Measurement of vitreous depth was significantly greater in the Holstein calves on the vertical scans (*p* ≤ 0.05). Measurement of corneal thickness and lens thickness with the LgT differed on the vertical scans (*p* ≤ 0.05).

### 3.5. Non-Valuable Parameters

A total of 320 measurements with each transducer were taken in horizontal and vertical scans. Very few parameters could not be measured with the LT on horizontal (four out of 320, 0.01%) and vertical scanning (five out of 320, 0.01%) or with the LgT on horizontal (two out of 320, 0.006%) and vertical scanning (eight out of 320, 0.02%), whereas far more parameters could not be measured with the CxT (*p* < 0.01) on horizontal (77 out of 320, 24%) and vertical scanning (85 out of 320, 26%). A comparison between the two breeds showed that many parameters could not be measured on horizontal (33 out of 160, 0.20%; 44 out of 160, 0.27%), respectively, and on vertical scanning (32 out of 160, 0.20%; 49 out of 160, 0.30%), respectively.

## 4. Discussion

In this study, the performance of three different transducers for ocular ultrasonography in healthy Holstein and Piedmontese calves in field conditions were compared. To our best knowledge, few studies have been conducted in cattle though the technique has proven a very useful tool for eye examination in human and veterinary medicine. In dogs and cats, for example, ocular ultrasound is a common diagnostic tool [8,9] and eye biometry has been widely studied [3,10,11].

**Figure 10:**
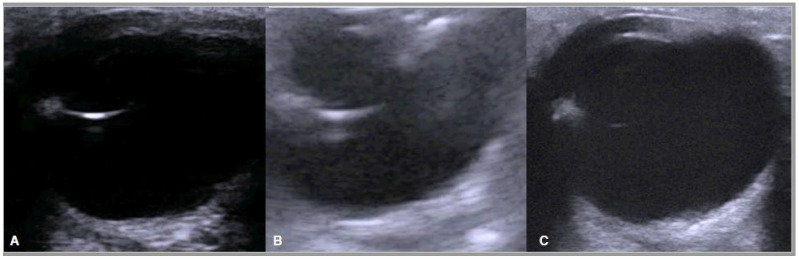
Same eye examined with three transducers: Linear (**A**), Convex (**B**), Linear Gynecological (**C**).

As reported in other studies, our results show how the eye structures could be visualized through ultrasonography [10], even if the different probes return different images (Figure 10).

Our biometry findings showed some difference from other studies [3,10]. Indeed, in both Holstein and Piedmontese groups, all the parameters, except for CT and SRT were lower than literature findings. It must be considered that the animals enrolled in other studies were adults of different breeds (Holstein, Jersey, Angus) so the difference could be due to an age effect.

It cannot be excluded that, evaluating the same animals enrolled in this study once they have become adults, the findings could be like the literature. Since several years have passed between the studies, such differences could also be related to the technological improvement and the different imaging of modern transducers compared to the older ones. Finally, those differences could be due to the eye anatomy and growth, so the CT and SRT could be structures poorly affected by the age. Moreover, with CxT, the SRT was higher than Potter reported [3], but the lower resolution of this probe, compared to the others, could probably have impaired this measurement.

However, it must be considered that in this study the US findings were not compared to gross anatomy since euthanasia could not be performed on healthy animals for ethical reasons and a certain bias relative to ultrasound examination itself cannot be completely ruled out. In the whole sample, no difference between LT and LgT measurement of biometric parameters was noted comparing vertical to horizontal scans, whereas differences in axial length, vitreous depth, corneal thickness, and optic nerve diameter were recorded with the CxT. In addition, the CxT did not allow for certain biometric measurements on the horizontal (77 out of 320, 24%) and the vertical (85 out of 320, 26.5%) scans, which might have been due to the poor adaptability of the transducer for ophthalmological use. Similarly, differences in measurement between the CxT and the LgT and the LT may be ascribed to probe design. The CxT, due to its lower MHz range, is more suitable for obtaining deeper scans. So, scanning superficial structures such as the eye could lead to altered or poorly detailed images [12].

Several significant differences in CxT measurement of biometric parameters in the Holstein calves were found and many parameters could not be measured with the CxT in the Piedmontese calves, which supports our hypothesis that optimal images of the eye cannot be obtained with the CxT, making it unsuitable for eye examination. Further comparison between the Holstein and the Piedmontese calves indicated that the differences between the two linear probes (LgT, LT) could be due to differences in eye anatomy of the two breeds. But here, too, a larger sample size is needed to address this hypothesis.

Our data show that the two linear probes allow for good visualization of eye structures, with the caveat that ultrasound examination is both operator- and device dependent. In addition, ultrasound had no negative effect on animal welfare. However, excessive pressure during maneuvering may compress the anterior structures of the eye, leading to underestimation of the biometric parameters of these structures or ocular damage. Finally, ultrasound exams performed with the LgT and the LT produced good quality images.

Overall, the results obtained are like those shown in dogs and cats [13]. However, unlike in pets [14,15,16,17], ultrasound studies relating to eye lesions are still few in bovine medicine. In pets, eye ultrasonography, is considered an adjunct to the ophthalmic examination, helps in reaching the correct diagnosis if the globe is opaque or the imaging or not visualizable structure is needed. Moreover, it often reduces the cost of more expensive imaging techniques [18]. Biometry measurement can be affected by several pathologies of the eye structures. As an example, the lens thickness measurement could differ between diabetic and normal dogs [5] and the globe axial length could be affected by intraocular masses or buphthalmia. Moreover, the biometry of the optic nerve can be affected by several pathologies, such as neuritis, intracranial hypertension, and diabetic ketoacidosis in both human and pets [19,20,21].

Our observations add to current knowledge in ocular ultrasonography, in which the LgT is widely used in field conditions. Therefore, the tools for the eye ultrasound examination are already available for the farm’s vet without further costs. Further studies are needed to establish evidence in eye conditions and to inform decisions in the diagnosis and treatment of eye disease in calves.

## 5. Conclusions

Ocular ultrasonography is a useful technique for eye examination in the field. While the LT and LgT transducers may not be as accurate as those for specialist ophthalmologic examination, they allow for an assessment of eye biometrics under field conditions. Given their widespread application in field practice and the ease of use of LgT, this transducer may provide a useful tool for eye examination in calves under field conditions. Further studies are needed to investigate this technique’s potential in diagnosing ocular disease.

## Figures and Tables

**Table 1:** Biometric parameters in the Holstein calves (n = 10) measured with three different transducers on horizontal and vertical scans.

	Linear Transducer		Convex Transducer		Linear Gynecological Transducer	
Parameter	Horizontal	Vertical	Horizontal	Vertical	Horizontal	Vertical
**Axial length (cm)**	2.5 ± (0.1)	2.5 ± (0.1) ^C^	2.4 ± (0.1) ^L^	2.4 ± (0.1) ^C,Q^	2.5 ± (0.2) ^L^	2.5 ± (0.1) ^Q^
**Anterior chamber depth (mm)**	2.5 ± (0.2)	2.6 ± (0.3) ^H^	2.3 ± (0.5)	2.2 ± (0.6)	2.5 ± (0.4)	2.4 ± (0.3) ^H^
**Vitreous depth (cm)**	1.3 ± (0.1) ^A^	1.3 ± (0.1)	1.2 ± (0.1) ^A^	1.3 ± (0.1)	1.3 ± (0.2)	1.3 ± (0.1)
**Corneal thickness (mm)**	1.1 ± (0.1) ^A,F^	1.0 ± (0.1) ^D,I^	1.6 ± (0.6) ^a,B,M^	1.1 ± (0.2) ^a,D^	1.2 ± (0.2) ^C,F,M^	1.1 ± (0.1) ^I^
**Scleroretinal rim thickness (mm)**	1.8 ± (0.2) ^A^	1.7 ± (0.3) ^E^	2.5 ± (0.4) ^B,N^	2.5 ± (0.3) ^E,R^	1.8 ± (0.2) ^N^	1.7 ± (0.2) ^R^
**Lens thickness (mm)**	7.7 ± (0.7)	7.7 ± (0.6)	7.7 ± (0.2)	7.6 ± (0.9)	7.7 ± (0.7)	8.0 ± (0.8)
**Lens length (cm)**	1.1 ± (0.1)	1.2 ± (0.1)	1.3 ± (0.1) ^b,O^	1.2 ± (0.1) ^b^	1.1 ± (0.2) ^O^	1.2 ± (0.1)
**Optic nerve head diameter (mm)**	5.6 ± (0.5) ^G^	5.7 ± (0.7)	7.1 ± (1.1) ^P^	6.6 ± (2.0)	6.1 ± (0.6) ^G.P^	6.1 ± (0.8)

**Same lowercase letters**: statistical differences between the same parameter on the horizontal and the vertical scan. **Same Uppercase letters**: statistical differences between the same parameter measured with different probes.

**Table 2:** Biometric parameters in the Piedmontese calves (n = 10) measured with three different transducers on horizontal and vertical scans.

	Linear		Convex		Linear Gynecological	
Parameter	Horizontal	Vertical	Horizontal	Vertical	Horizontal	Vertical
**Axial length (cm)**	2.4 ± (0.1) ^A^	2.4 ± (0.1) ^F^	2.2 ± (0.2) ^b,A,O^	2.3 ± (0.2) ^b,F,S^	2.4 ± (0.2) ^O^	2.4 ± (0.2) ^S^
**Anterior chamber depth (mm)**	2.6 ± (0.6)	2.7 ± (0.2)	2.6 ± (0.3)	2.4 ± (0.5)	2.4 ± (0.3)	2.5 ± (0.2)
**Vitreous depth (cm)**	1.3 ± (0.1) ^B^	1.3 ± (0.1) ^G^	1.2 ± (0.1) ^B,P^	1.2 ± (0.1) ^G,T^	1.3 ± (0.1) ^P^	1.1 ± (0.1) ^T^
**Corneal thickness (mm)**	1.1 ± (0.1) ^C,M^	1.1 ± (0.2) ^H,N^	1.4 ± (0.5) ^C^	1.2 ± (0.2) ^H^	1.2 ± (0.3) ^M^	1.2 ± (0.2) ^N^
**Scleroretinal rim thickness (mm)**	1.8 ± (0.3) ^a,D^	2.0 ± (0.2) ^a,I,O^	2.8 ± (0.5) ^D,Q^	3.0 ± (0.6) ^I,U^	1.8 ± (0.2) ^Q^	1.8 ± (0.2) ^O,U^
**Lens thickness (mm)**	7.5 ± (1.6)	7.6 ± (1.1)	7.1 ± (1.3)	7.2 ± (1.0)	7.7 ± (1.3)	7.3 ± (1.2)
**Lens length (cm)**	1.1 ± (0.1)	1.1 ± (0.2)	1.2 ± (0.2)	1.2 ± (0.1)	1.1 ± (0.2)	1.2 ± (0.1)
**Diameter of optic nerve head (mm)**	5.8 ± (0.6) ^E^	5.8 ± (0.9) ^L^	7.0 ± (1.3) ^E,R^	7.0 ± (1.0) ^L,V^	5.9 ± (0.8) ^R^	6.3 ± (0.7) ^V^

**Same lowercase letters** denote statistical differences between the same parameter on the horizontal and the vertical scans. **Same Uppercase letters** denote statistical differences between the same parameter measured with different probes.

## Data Availability

All data used in the current study are available from the corresponding author on reasonable request.

## Supplementary Materials

The following supporting information can be downloaded at: https://www.mdpi.com/article/10.3390/ani13040742/s1, Supplementary File: Table S1.
